# VGF Changes during the Estrous Cycle: A Novel Endocrine Role for TLQP Peptides?

**DOI:** 10.1371/journal.pone.0108456

**Published:** 2014-10-03

**Authors:** Barbara Noli, Carla Brancia, Filomena D’Amato, Gian-Luca Ferri, Cristina Cocco

**Affiliations:** Department of Biomedical Sciences, University of Cagliari, Monserrato (Cagliari), Italy; University of Rouen, France, France

## Abstract

Although the VGF derived peptide TLQP-21 stimulates gonadotropin-releasing hormone (GnRH) and gonadotropin secretion, available data on VGF peptides and reproduction are limited. We used antibodies specific for the two ends of the VGF precursor, and for two VGF derived peptides namely TLQP and PGH, to be used in immunohistochemistry and enzyme-linked immunosorbent assay complemented with gel chromatography. In cycling female rats, VGF C-/N-terminus and PGH peptide antibodies selectively labelled neurones containing either GnRH, or kisspeptin (VGF N-terminus only), pituitary gonadotrophs and lactotrophs, or oocytes (PGH peptides only). Conversely, TLQP peptides were restricted to somatostatin neurones, gonadotrophs, and ovarian granulosa, interstitial and theca cells. TLQP levels were highest, especially in plasma and ovary, with several molecular forms shown in chromatography including one compatible with TLQP-21. Among the cycle phases, TLQP levels were higher during metestrus-diestrus in median eminence and pituitary, while increased in the ovary and decreased in plasma during proestrus. VGF N- and C-terminus peptides also showed modulations over the estrous cycle, in median eminence, pituitary and plasma, while PGH peptides did not. In ovariectomised rats, plasmatic TLQP peptide levels showed distinct reduction suggestive of a major origin from the ovary, while the estrogen-progesterone treatment modulated VGF C-terminus and TLQP peptides in the hypothalamus-pituitary complex. In *in vitro* hypothalamus, TLQP-21 stimulated release of growth hormone releasing hormone but not of somatostatin. In conclusion, various VGF peptides may regulate the hypothalamus-pituitary complex *via* specific neuroendocrine mechanisms while TLQP peptides may act at further, multiple levels *via* endocrine mechanisms involving the ovary.

## Introduction

The VGF gene product, and/or its derived peptides, appear to be involved in reproduction since *vgf* null mice were sexually immature and almost completely infertile [1]. The 66 kDa VGF precursor [1]–[6] is composed of 617 or 615 amino acid residues (in rat or human, respectively), and gives rise to several low molecular weight VGF peptides which are abundant in multiple brain regions, peripheral neurones, and certain endocrine and neuroendocrine cell populations [7]–[13]. Despite their abundance and wide distribution, limited data are available on their role and function/s. Among the VGF peptides with proven biological activity are included TLQP-21 [14], TLQP-62 [15] and the peptides called NERPs [16]. TLQP-21 was shown to act on various mechanisms, including the regulation of energy balance [14], inflammatory and neuropathic pain [17], [18], chronic stress [19], and gastric motility and emptying [20]. With respect to reproduction, induction of VGF mRNA was reported in the pituitary immediately after the estrus, in parallel with a clear-cut decrease in certain VGF peptides, as well as changes in their localisation in gonadotrophs and lactotrophs [21]. A distinct seasonal modulation in cell-type-specific processing of the VGF precursor was revealed in the anterior pituitary of female sheep [22], while significant upregulation of VGF mRNA was found related to reproductive maturation in baboon ovary [23]. More recently, TLQP-21 was shown to exert a number of actions on the rat reproductive axis [24], [25]. Central administration of TLQP-21 in pubertal and adult male rats induced gonadotrophin secretion *via* release of gonadotropin-releasing hormone (GnRH), and stimulated testosterone secretion *in vitro* in pre-pubertal animals [24]. In female pre-pubertal rats, TLQP-21 induced *in vitro* secretion of luteinising hormone (LH) and follicle-stimulating hormone (FSH) from the pituitary, with no detectable effect on GnRH release from the hypothalamus [25]. On the same rats, upon *i.p*. injection of the same peptide at high doses, serum LH showed a moderate increase, while after central administration of TLQP-21 in adults animals, the LH response was dependent on the stage of the reproductive cycle [25]. On the whole, TLQP-21 would so far appear to affect female reproduction by stimulating pituitary LH release. We produced antisera selective for the C-and N-terminal portions of the VGF precursor, and for two cleaved peptides “TLQP” and “PGH” (named from the corresponding four N-terminal, and three C-terminal amino-acids, respectively, immediately adjacent to their respective sites of cleavage from the VGF precursor), to be used for immunohistochemistry (IHC) and enzyme-linked immunosorbent assay (ELISA), complemented with gel chromatography. We addressed the localisation and changes of VGF peptides in the female rat reproductive axis in connection with the estrous cycle, as well as after ovariectomy. Their presence and modulation in plasma was outlined in parallel, as a clue to their possible endocrine significance. In addition, in view of its selective distribution in the hypothalamus, the ability of the TLQP-21 peptide to release somatostatin or growth-hormone-releasing hormone (GHRH) was tested *in vitro*.

## Materials and Methods

### Animals and tissue samples

Sprague Dawley adult female rats (from Charles River, Italy, 200–250 g body weight) were kept at 22°C (12∶12-h light/dark cycle, lights on 7 am–7 pm), 3 to 4 rats *per* cage, with food and water *ad libitum*. All animals were killed at the same time of day (approximately 10 am), with a diethyl ether overdose. Group 1 (“cycling rats”) included females showing regular 4 days estrous cycles (vaginal smears were taken daily at 8.30–9.30 during about 2 consecutive cycles, on average). Rats were killed at the age of 12–14 weeks, at proestrus, estrus, diestrus, or metestrus (n = 12 *per* each cycle phase). Group 2 rats (“ovariectomy + estrogen/progesterone treatment”, n = 8) were bilaterally ovariectomised at 3–4 weeks’ age, hence received intraperitoneal beta-estradiol (200 ug/200 ul, 2 days before sacrifice) and progesterone (500 ug/200 ul, on the day of sacrifice). Group 3 rats (“ovariectomy controls”, n = 7) were as for group 2, but received the corresponding solvent only (seed oil). Rats from groups 2 and 3 were sacrificed in parallel, 4 hours after the progesterone/oil injection. All experimental protocols were approved by the Ethical Committee at the University of Cagliari, and were performed in accordance with the guidelines for the care and use of animals approved by the American Physiological Society, the EEC Council Directive of 24 November 1986 (86/609) and the relevant Italian legislation. For ELISA experiments, the brain, hypophysis and ovaries were extracted from each rat as follows: group 1, n = 8 *per* phase; group 2, n = 5; group 3, n = 4. For group 1, the median eminence was dissected (under a stereomicroscope) from the remainder of the hypothalamus, and the two were separately extracted. The whole hypothalamus, inclusive of the median eminence, was taken from rats of groups 2 and 3. Blood (approximately 5 ml) was drawn at sacrifice (hence for cycling rats at the morning of each cycle phase as above mentioned) from the left heart of every animal, collected in an EDTA (1.78 mg/ml) containing tube, and rapidly centrifuged (3,000× *g*, 10–15 minutes), hence plasma was stored frozen. Tissues were extracted in ice-cold phosphate buffered saline (PBS, 0.01 mol/l PO_4_, pH 7.2–7.4, 0.15 mol/l NaCl: 10 ml/g tissue) containing protease inhibitor cocktail (P8340, Sigma-Aldrich, Schnelldorf, Germany), homogenized for 3 minutes using an ultra Turrax (Ika-Werke, Staufen, Germany), hence tubes were heated in a vigorously boiling water bath for 10 minutes, and centrifuged (3,000×*g*, 10 minutes). Supernatants were stored frozen until use (−20°C or lower). For IHC, rats (group 1, n = 4; groups 2 and 3, n = 3 each) were transcardially perfused with 4% paraformaldehyde (in 0.2 mol/l PO_4_ buffer, pH = 7.2), hence brain, pituitary and ovary were dissected out, and rinsed overnight in PBS containing 7% sucrose. Samples were oriented in aluminum foil moulds with cryoembedding media [26], snap-frozen and stored in a liquid nitrogen tank (vapour phase). Sections (5–7 um) were obtained using a cold-blade cryomicrotome [27] (Microm HM-560, Walldof, Germany), and collected on poly-L-lysine treated slides.

### Antibodies

**Figure 1:**
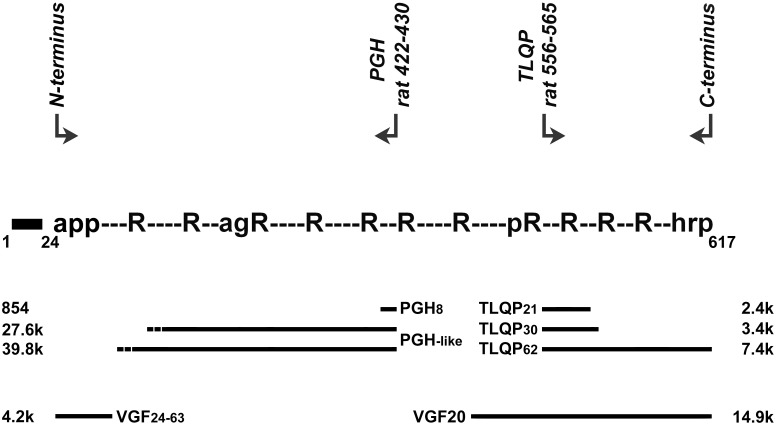
The VGF precursor, amino acid stretches used to raise antibodies, and known/putative VGF peptides. Top panel: arrows are aligned with 9 or 10 amino acid stretches used to raise antibodies, directed towards the site of conjugation with carrier protein. Middle panel: along the primary sequence of VGF (not drawn to scale) are shown: dibasic cleavage sites (R, or: pR) and a cleavage-amidation site (agR), the N- and C-terminal ends of the VGF precursor (“app-“ and “-hrp”, respectively: single letter notation of the corresponding amino acids), and the signal peptide (1–24 black box). Antibodies raised against VGF C-or N-terminus peptides are expected to react with any peptide cleaved from VGF, which includes the corresponding sequence. Antibodies to PGH and TLQP are expected to react well with any peptide extended on the “conjugation” side (N-terminal side of PGH, and C-terminal side of TLQP, respectively). Bottom panel: several known VGF peptides (relevant to the present study) together with their MW are aligned with the precursor. “PGH-like” peptides are intended to outline two putative peptides, running from: the PGH sequence, to: the N-terminal VGF_24–36_ peptide for the 39.8 kDa form, or to: a cleavage site (rat VGF_178–179_) implicated in the biosynthesis of NERP-3 for the 27.6 kDa peptide. Their hypothetical N-terminal extreme is pointed out (dotted bar).

The antibodies used are summarized in Table 1. VGF peptides used for immunizations (Fig. 1) encompassed: (i) the rat VGF C-terminal nonapeptide; (ii) the N-terminal decapeptide of the rat VGF precursor (VGF24-33, upon cleavage of the rat VGF1-23 signal peptide); (iii) the N-terminal decapeptide of rat TLQP peptides (rat VGF556-565), and: (iv) the C-terminal nonapeptide of PGH peptides (rat VGF422-430). These were conjugated with bovine thyroglobulin or keyhole limpet haemocyanin, via either an additional N-terminal D-tyrosine residue (for VGF C-terminus and PGH), or an additional C-terminal cysteine (for VGF N-terminus and TLQP), as previously described (Tables 1–2). Hormone and hypothalamic factor antibodies (Table 1) showed negligible (<0.5%) cross reactivity with other pituitary hormones [21], [22].

**Table 1:** Antisera and antibodies used in the study.

	Antigen	Specie	Ref.	Dilutions	use
**VGF peptides**	C-t.	Rabbit	Ferri *et al.* 1995	1∶250 K/1∶3 K	E/I
			Cocco *et al.* 2007		
	TLQP	G. Pig	Brancia *et al.* 2005	1∶60 K/1∶1000	E/I
			D’Amato *et al.* 2008		
	PGH	Rabbit	D’Amato *et al.* 2008	1∶60 K/1∶1000	E/I
	N-t.	G. Pig	Cocco *et al.* 2010	1∶400 K/1∶800	E/I
**Hormones**	FSH	Mouse	Berger *et al.* 1990	1∶500	I
	GH	Monkey	AF Parlow	1∶600	I
	LH	Sheep	Biomol/Enzo	1∶600	I
	PRL	Mouse	Berger *et al.* 1990	1∶600	I
	GHRH	Rabbit	Phoenix	1∶16000	E
	GHRH	GHRH Rabbit	Phoenix	1∶6000	E
**h. factors**	GnRH	Mouse	Sternb	1∶2000	I
	SRIF	Rabbit	Abcam	1∶20 K/1∶600	E/I
	Kissp	Rabbit	Millipore	1∶800	I

C-term: VGF C-terminus; N-term: VGF N-terminus; FSH: follicle-stimulating hormone; GH: growth hormone; LH: luteinising hormone; PRL: prolactin; h. factor: hypothalamic factor, GHRH: growth hormone-releasing hormone; GnRH: gonadotrophin-releasing hormone; SRIF: somatostatin, Kissp: kisspeptin; Ref: references; E: ELISA; I: immunohistochemistry.

**Table 2:** ELISA characterization.

	Peptide	Sequence	IC_50_	CV1	CV2	% cross- reactivity
	rVGF_609–617_^2^	**––dY-IEHV LLHRP**				100
**C-t**	hVGF_607–615_	**IEHV LLRRP**	0.02	2–3	4	<0.01
	hVGF_603–612_	**LEN YIEHV LL**				<0.01
						
	rVGF_556–565_^2^	**TLQPP ASSRR–C––**				
	rVGF_556–564_	**TLQPP ASSRR**				100
**TLQP**	rVGF_556–567_	**TLQPP ASSRR R**	1.1	3–5	6	122
	rVGF_556–576_	**TLQPP ASSRR** **RHFHH ALPPA R**				183
	rVGF_556–564_-R^3^	**R-TLQPP ASSR**				3.5
						
	rVGF_422–430_^2^	**–dY-RSQEE APGH**				100
**PGH**	hVGF_422–431_-R^4^	**RSQEE APGH-R**	0.3	4–6	6–8	<0.015
	hVGF_419–427_	**RSQEE TPGH**				3.5
	hVGF_419–428_-R^4^	**RSQEE TPGH-R**				<0.015
						
	rVGF_24–31_^2^	**APPGR SDVYP-C––**				100
**N-t**	rVGF_23–30_	**APPGR PEAQP**	10	3–7	5–8	<0.001
	mVGF_24–31_	**APPGR PDVFP**				<0.001

Sequence differences versus rat VGF are underlined; IC50: dose producing 50% inhibition (pmol per well); CV1 and CV2: intra and inter assay variation, respectively, C-t and N-t: VGF C-terminus and N-terminus, respectively; ^2^ “immunogen”: peptide used for conjugation to carrier protein and immunizations; used for plate coating and assay standards; R^3^: Arg-extended: with addition of Arginine residue at the peptide N- or C-terminus, respectively; R^4^ additional Arginine residue from adjacent cleavage site; “dY” and “C”: additional d-Tyrosine, or Cysteine residue used for conjugation; r: rat, h: human, m: mouse.

### Characterization of VGF antibodies

Specificity was addressed in parallel *via* immunohistochemistry and ELISA. In the former, labeling with each VGF antibody was prevented by pre-absorbed with the relevant peptide (up to 50 nmol/ml). At the C-terminus of VGF, the rat sequence shows a single amino acid difference *versus* human VGF (Table 2). Both the human nonapeptide and other peptide missing the last three C-terminal amino acids (HVLL) showed negligible reactivity in ELISA (Table 2), while in IHC the rat VGF C-terminus antibody showed: (i) low reactivity on human tissues; (ii) clear-cut no-colocalisation of VGF C-terminus and HVLL immunostaining [28]. On such basis, the latter antibody is considered to recognise a short intact sequence at the extreme C-terminus of rat VGF. Species differences are shown in the VGF N-terminal decapeptide (Table 2), with very low reactivity of mouse and human forms in ELISA. Hence, antibody reactivity would depend on a comparatively long portion of the immunogen sequence. Cleaved peptides with an exposed TLQP or PGH sequence (i.e. with such sequence present at their N-terminus, and C-terminus, respectively) can be formed via proteolysis at the adjacent cleavage sequence: Arg_553_-Pro_554_-Arg_555_, for TLQP peptides [12], and: Arg_431_-Arg_432_, for PGH peptides. Synthetic peptides similar to immunogens, with the addition of one extra Arginine in the location of their respective cleavage sequence (“Arginine-extended”, see Table 2), were used to gain insight into the potential cross-reactivity of antibodies with native extended peptides (these may include the whole VGF precursor, or any peptides cleaved from VGF and including the TLQP or PGH sequence internally). In ELISA, the coating of specific peptide antigen onto the wells, together with high antibody dilutions, results in selection of the antibodies taking part in the reaction, hence in a major increase in specificity for cleaved peptides. Furthermore, most native peptides will be extended by many more than the one (Arginine) residue we tested, thus favouring low cross-reactivity.

### Immunostaining

Sections were incubated overnight in a humid chamber, with antibodies diluted in PBS containing 30 ml/l of normal donkey serum, 30 ml/l of normal rat serum, and 0.02 g/l NaN_3_. Double and triple immunofluorescence experiments were carried out mixing a VGF antibody with one hormone or hypothalamic factor antibody (Table 1) obtained in a different donor species. The relevant species-specific secondary antibody/ies (all from donkey, affinity purified IgG preparations absorbed with serum proteins from multiple species, conjugated with either Cy_3_, Cy_2_ or AMCA: Jackson Immunoresearch Laboratories, West Grove, PA) were used to reveal the sites of primary antibody binding. Slides were coverslipped with PBS-glycerol, observed and photographed using BX41 and BX51 fluorescence microscopes (Olympus, Milan, Italy) equipped with Fuji S2 and S3 Pro digital cameras (Fujifilm, Milan, Italy). Further to absorption controls (see above), routine controls included substitution of each antibody, in turn, with PBS, the use of pre-immune or non-immune sera, and the testing of each secondary antibody with their respective non-relevant primary antibodies.

### ELISA

VGF assays were carried out as described previously [28]–[29]. Briefly, multiwell plates were coated with either the relevant VGF peptide (Table 2), or synthetic GHRH (Bachem, Bubendorf, Switzerland, 120 pmol/ml), as appropriate, and blocked with PBS containing 9% normal donkey serum and 0.05% NaN_3_ (2 h). Primary incubations were carried out with a mixture of the relevant primary antibody (in PBS containing 9% normal donkey serum, 20 nmol/L aprotinin, 1 mg/ml EDTA and 0.05% NaN_3_), and either samples (diluted 1∶5 to 1∶40), or serial dilutions of the relevant standard peptide, or the relevant peptides to be tested for characterization purposes (Table 2). Detection was carried out with the relevant secondary antibody (affinity purified donkey IgG, absorbed with serum proteins from multiple species, biotin conjugated: 1 h, from: Jackson), streptavidin-peroxidase conjugate (Biospa, Milan, Italy: 30 min), and tetramethylbenzidine substrate (TMB X-tra, Kem-En-Tec Diagnostics, Taastrup, Denmark). Optical density was measured at 450 nm using a multilabel plate reader (Chameleon, Hidex, Turku, Finland). For the somatostatin assay, plates were coated with donkey anti-rabbit IgG (as above, 1∶3000), blocked as above, and incubated with a mixture of somatostatin antibody (diluted in the above medium), biotin-conjugated somatostatin (Bachem, 100 pmol/ml), and either samples of heat-treated culture medium (diluted 1∶10), or serial dilutions of unconjugated somatostatin (Bachem). Detection was carried out with streptavidin-peroxidase, as above. VGF assay characterization is summarised in Table 2. GHRH and somatostatin assays showed low intra- and inter-assay variation (CV%<10%).

### Chromatography

Extracts (2 ml) were individually loaded onto a Sephadex G-50S column (Sigma; 2 cm^2^×1 m), equilibrated with 50 mM ammonium bicarbonate and eluted with the same buffer. A molecular weight (MW) marker kit (MWGF70: Sigma) was used for column calibration. Fractions (3 ml) were reduced in volume with a Vacufuge Concentrator (Eppendorf, Milan, Italy) and assessed by ELISA. For all VGF peptides studied, overall recovery of loaded immunoreactivity ranged between 80% and 100%.

### *In vitro* experiments

In view of the specific localisation of TLQP peptides in median eminence somatostatin-containing terminals, we hypothesized a possible action onto secretion of somatostatin and its GHRH neurone targets. Adult male rats (Sprague Dowley, 300–350 g body weight, n = 12) were killed by decapitation, and their brain was rapidly dissected out. Each hypothalamus (from optic chiasm to mammilary bodies, about 2 mm wide) was incubated in Dulbecco’s modified Eagle’s medium (DMEM, with 4.5 g/L glucose, without glutamine) in a 95% O_2_ and 5% CO_2_ atmosphere, at 37°C, as previously described [24]. After 30 min, the medium was removed and substituted with fresh medium with/without TLQP-21 (10^−6 ^mol/L; n = 6 and 6 hypothalami). After a further 45 minutes incubation, culture media were collected, heat treated in a boiling water bath for 30 min, and stored frozen pending somatostatin and GHRH assays.

### Statistical analysis

Data were expressed as mean ± SEM throughout. Statistical analyses were carried out by one-way ANOVA, followed by *t*-test as appropriate (StatistiXL software, www.statistixl.com), and a *P*<0.05 was considered statistically significant.

## Results

### VGF peptide immunolocalisation

**Figure 2:**
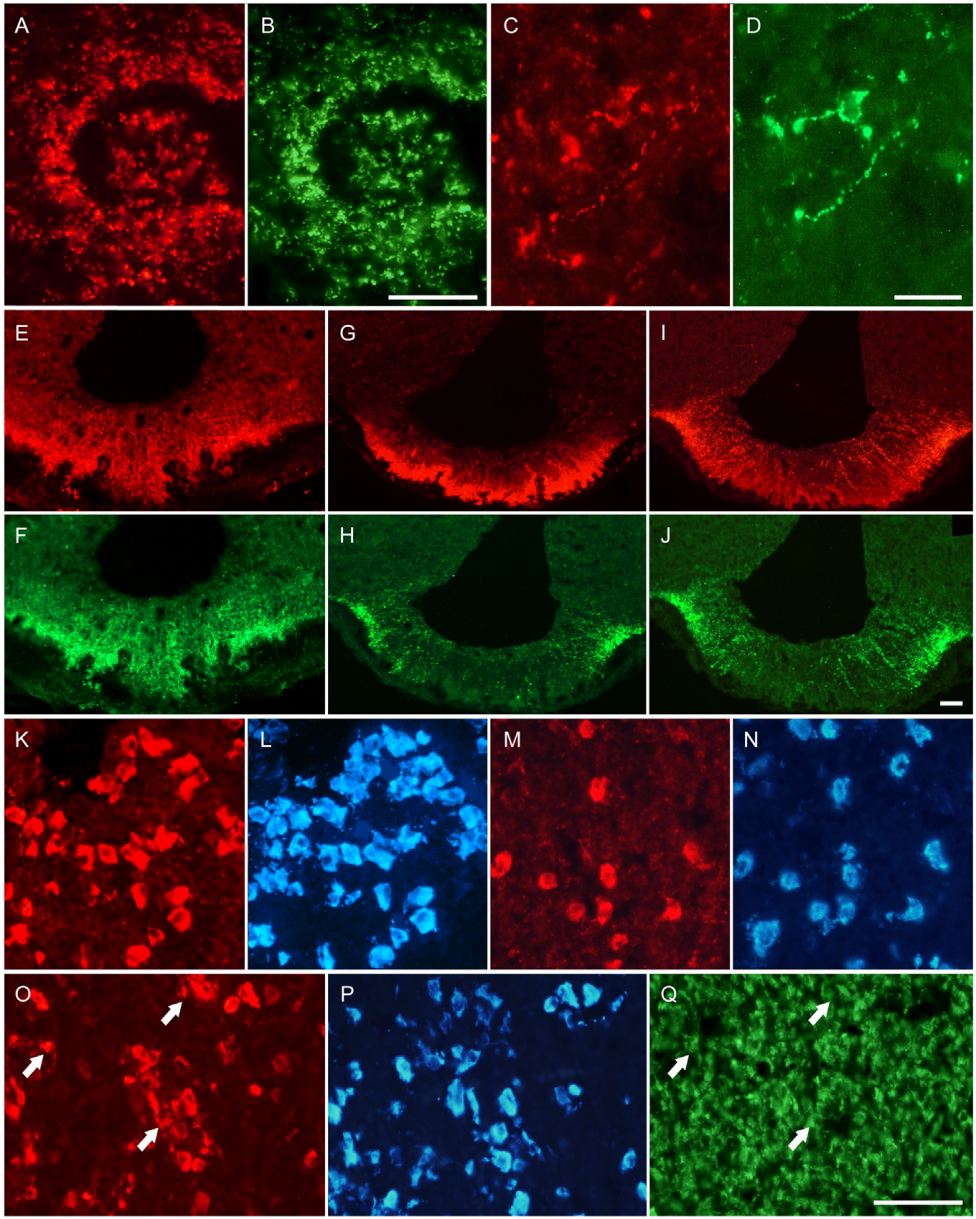
VGF peptide localisation in hypothalamus and pituitary. In the preoptic area (A–D), PGH peptides (A) were immunostained within almost all neuronal terminals containing GnRH (B) conversely, VGF N-terminus peptides (C) were also revealed in most kisspeptin neuronal terminals (D): A, C: Cy3 red labeling; B, D: Cy_2_ green labeling. In the median eminence (E–J), TLQP peptides (E) were selectively found in almost all somatostatin neuronal terminals and axons (F). Conversely, VGF C-terminus (G) and PGH (I) antibodies labeled GnRH neurone terminals (H and J, respectively): E, G, I: Cy3 red labeling; F, H, J: Cy_2_ green labeling. In the pituitary (K–Q), the number of cells positive with both TLQP (K, M) and LH (L, N) antibodies was higher in diestrous (K, L) than in estrous phase (M, N). Conversely, the PGH peptides (O) were found in a large number of LH cells (P) and in a few PRL cells (Q, colocalised cells are identified by the arrows). Scale bars: A–B, E–Q: 100 um, C–D: 50 um.

**Figure 3:**
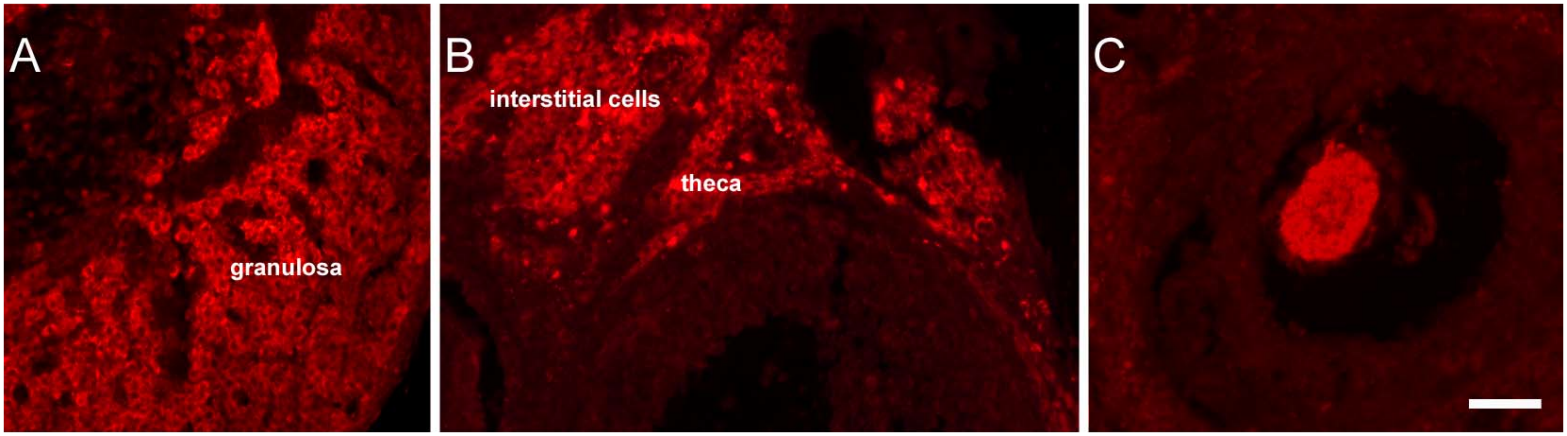
VGF peptide localisation in Ovary. TLQP peptide immunoreactivity was found in the granulosa (A) as well as in the theca and interstitial cells (B), while PGH peptides were restricted to the oocyte (C). All VGF staining: Cy_3_ red labeling. Scale bar: 50 um.

Immunoreactive VGF peptides were revealed in multiple areas of the reproductive axis as hypothalamus, including the preoptic area (POA) and median eminence, as well as in hypophyseal and ovarian cells (Fig. 2 and 3). Experiments focussed on the POA showed PGH peptide immunoreactivity in the majority of GnRH axons and terminals (Fig. 2, A and B), with weak labeling of some GnRH containing perikarya (not shown). The same applied to VGF C-and N-terminus antibodies, with the latter additionally labeling almost all kisspeptin containing fibres (Fig. 2C and D). In the median eminence, TLQP peptides were brightly immunostained (Fig. 2, E), confined to somatostatin containing axons and terminals (Fig. 2, F). VGF C-and N-terminus peptides were far more widely distributed throughout the external and internal median eminence, including axons and terminals containing GnRH (Fig. 2, G and H), or kisspeptin (VGF N-terminus only, not shown). PGH peptides showed a discrete localisation, in the lateral portion of the median eminence, virtually completely colocalised with GnRH (Fig. 2, I and J). The above distribution profiles of VGF peptides in the POA and median eminence remained broadly similar over the estrous cycle (not shown). In the anterior pituitary (Fig. 2, K–Q), all VGF antisera labelled large numbers of endocrine cells. The majority (80–90%) proved to be LH containing gonadotrophes (Fig. 2, K–N and O, P: TLQP and PGH peptides, respectively). A small proportion (about 10–20%) of VGF C- or N-terminus, or PGH immunoreactive cells (Fig. 2, O, Q) were prolactin containing lactotropes. In the morning following estrus, immunostaining of gonadotropes (especially with VGF C-and N-terminus and TLQP antibodies) was reduced, with an increase of visible labeling in a fraction of lactotropes, as shown previously [21] (Fig. 2, K, L *versus* M, N: diestrus *versus* estrus, respectively).

In the ovary (Fig. 3), TLQP peptide immunoreactivity was revealed in the granulosa (Fig. 3A) as well as in the theca and interstitial cells (Fig. 3B). Conversely, PGH peptide/s were restricted to oocytes (Fig. 3, C), and VGF C- and N- terminus peptide labeling was scanty. In ovariectomised rats, most hypertrophic pituitary gonadotropes showed VGF peptide immunoreactivity, with intense to moderate labeling intensity, as reported previously [21].

### Changes in cycling females

As with any other quantitative method based on the use of antibodies, the numerical values of peptide concentrations measured in ELISA ought to be considered with caution, pending sequencing of the relevant native peptide/s, hence use of proper standards.

**Figure 4:**
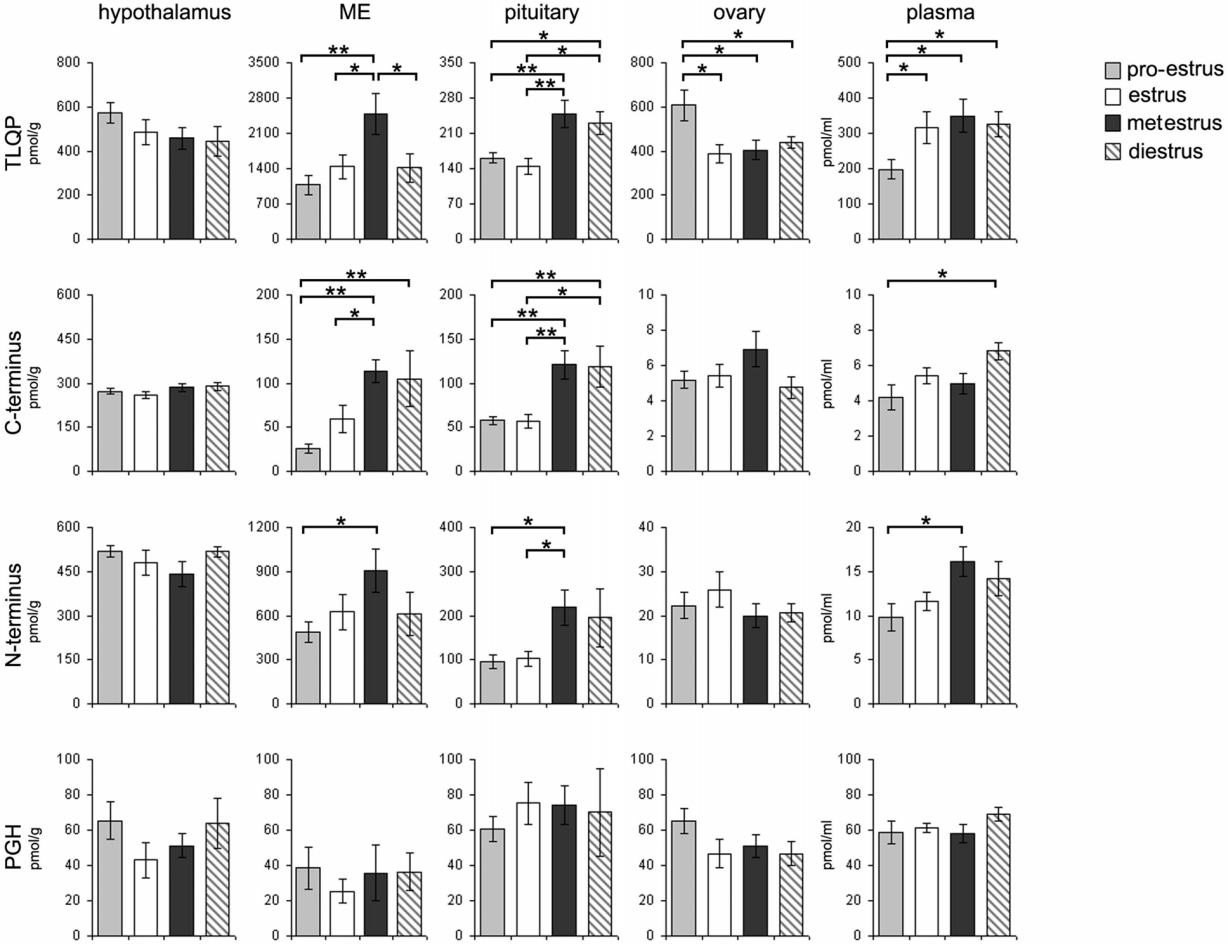
VGF peptide levels in cycling females. Levels of TLQP, C-/N- terminus and PGH peptides were measured in hypothalamus, median eminence (M.E), pituitary, ovary and plasma in each phase of the estrous cycle. During metestrus and/or diestrus, enhancement was revealed for TLQP and the C-/N-terminal peptides in M.E, pituitary and also in plasma. In the pro-estrous phase, exclusively TLQP peptides were found to be increased in ovary and decreased in plasma. PGH peptides did not show any change. Pmol/g: picomoles per grams; pmol/ml: picomoles per millilitres; *p<0.05, **p<0.005.

Nonetheless, TLQP peptides appeared to be most abundant in all areas we studied, as well as in plasma (Fig. 4), with lowest levels for VGF C-and N-terminus peptides in ovary and plasma. Along the estrous cycle, TLQP levels were higher during metestrus in median eminence and during metestrus and diestrus in pituitary. The same peptides were more abundant in ovary and lower in plasma during proestrus, compared to the other phases. VGF C-and N-terminus peptides, too, increased during metestrus and/or diestrus in median eminence and pituitary, as well as in plasma. PGH peptides failed to show significant modulation.

### Ovariectomy and hormonal treatment

**Figure 5:**
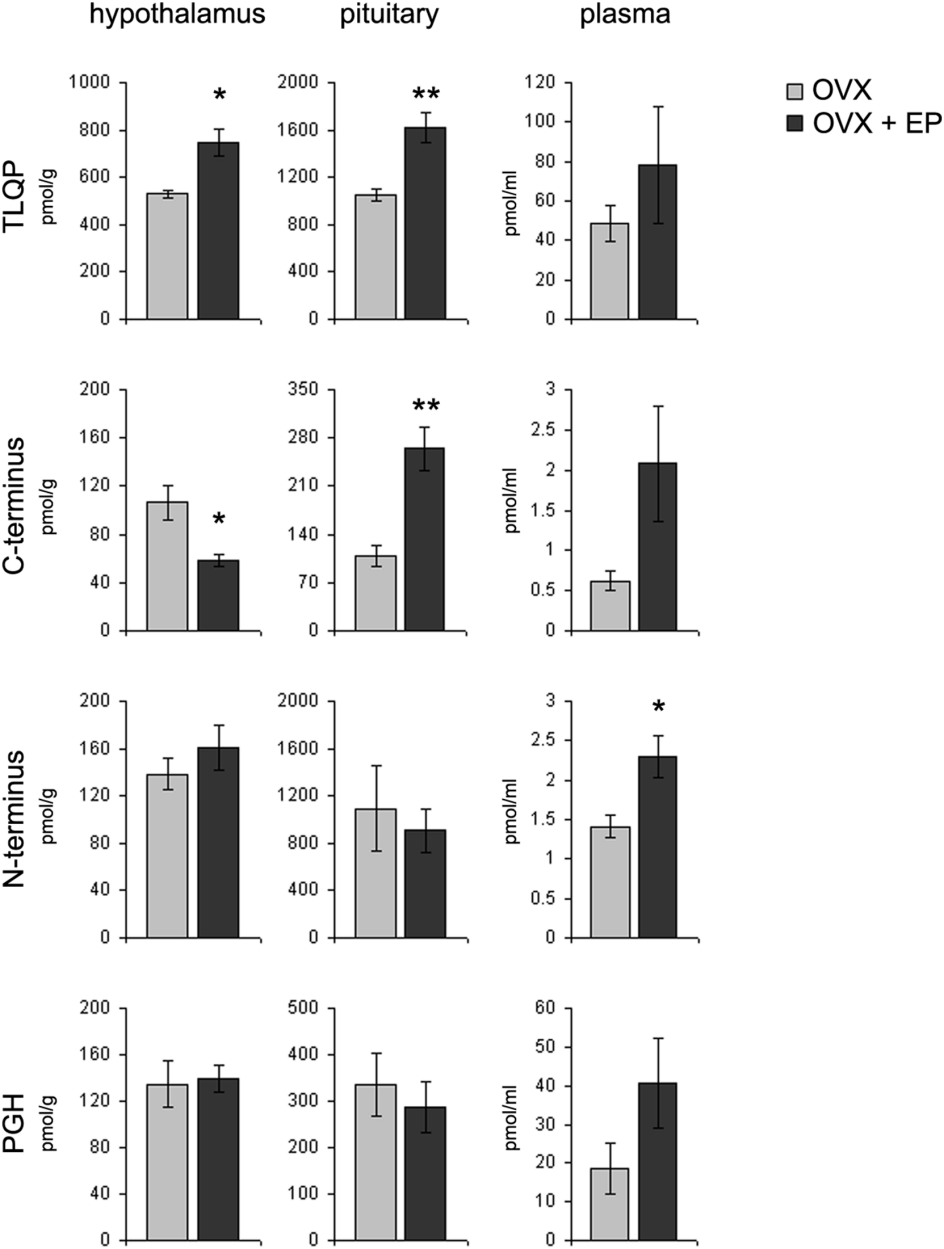
VGF peptide levels in ovariectomised (OVX) rats. Levels of TLQP, C-/N-terminus and PGH peptides were measured in the hypothalamus (including median eminence), pituitary, and plasma of OVX rats with and without oestradiol-progesterone (EP) treatment: OVX+EP and OVX, respectively. OVX+EP rats revealed an increase for TLQP peptides in hypothalamus and pituitary as well as changes for C-terminal peptides. In plasma, after EP treatment an increase was revealed for N-terminal peptides only. The PGH peptides did not reveal any change. Pmol/g: picomoles *per* grams; pmol/ml: picomoles per millilitres; *p<0.05, **p<0.005.

In the absence of hormonal treatment, upon ovariectomy TLQP peptides were greatly reduced in plasma (roughly 20% of average levels in cycling females: Figs. 4, 5). The other VGF peptides studied were also decreased, to very low levels. As measured in the “whole hypothalamus”, post-ovariectomy TLQP and PGH peptides were roughly comparable to cycling females, while VGF N- and C-terminus peptides were reduced (Figs. 4, 5). In the pituitary, most VGF peptides studied were distinctly increased, except for VGF C-terminus containing ones. Upon estrogen-progesterone treatment, a differential response was shown for TLQP and VGF C-terminus peptides, both further increasing in the pituitary, while in hypothalamus TLQP increased, and VGF C-terminus peptides were reduced (Fig. 5). In plasma, all VGF peptides studied showed a tendency to increase after hormonal treatment, with significant differences for VGF N-terminus peptides only.

### Molecular heterogeneity

**Figure 6:**
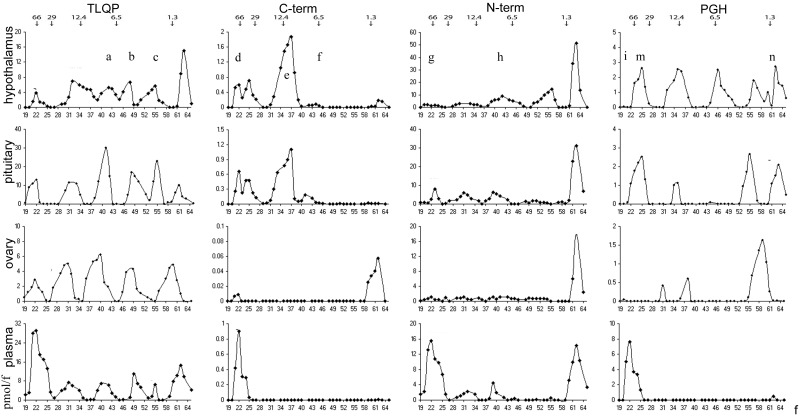
Sephadex chromatography. Three major peaks were shown for TLQP peptides (left: a, b, c), corresponding to TLQP-62, TLQP-30 and TLQP-21 and found in all tissue extracts. Higher MW forms were seen, at approximately 10–12 kDa and in the void volume, or in the region overlapping the void volume in plasma. VGF C-terminus immunoreactivity was revealed in the void volume (mid-left: d), compatible with VGF precursor, and in a major broad peak in the approximately 10–15 kDa range (ibidem: e), while a lower peak (ibidem: f) appeared related to the TLQP “a” peak described above. The same peptides were mostly seen as low MW fragments in the ovary, while migrating as large forms in plasma (mid-left). The VGF N-terminus assay revealed abundant reactivity in the large MW fractions from plasma (mid-right: refer “g” to bottom panel), with lower levels at varied MW in the other tissue extracts. PGH peptides were revealed as different MW forms, especially in the hypothalamus while the largest MW forms eluting in the void volume were abundant in plasma (ibidem: i). Molecular weight markers are shown with the corresponding arrows. Pmol/f:picomoles/fraction; f:fraction.

To resolve small to medium MW peptides we resorted to gel chromatography, with detection by ELISA in view of its enhanced selectivity for internally cleaved peptides (see above). Three major peaks were shown for TLQP peptides (Fig. 6, left: a, b, c), closely matching the elution positions shown previously for TLQP-62, TLQP-30 and TLQP-21 [30]. Such forms were found in all tissues studied and in plasma, with a lower TLQP-21 peak in the ovary. Higher MW forms were seen, at approximately 10–12 kDa and in the void volume, or in the region overlapping the void volume in plasma (Fig. 6, left). VGF C-terminus immunoreactivity was revealed in the void volume (Fig. 6, mid-left: d), compatible with VGF precursor, and in a major broad peak in the approximately 10–15 kDa range (ibidem: e), possibly representing previously described VGF18 or VGF20 [11], [12]. A lower peak (ibidem: f) appeared to relate to the TLQP “a” peak described above. The same peptides were mostly seen as low MW fragments in the ovary, while migrating as large forms in plasma (Fig. 6, mid-left). The VGF N-terminus assay also showed abundant reactivity in the large MW fractions from plasma (Fig. 6, mid-right: refer “g” to bottom panel), with lower levels at varied MW in tissue extracts. In the hypothalamus, the N-terminal VGF24–63 peptide [31] may take part in the lower MW portion (Fig. 6, mid-right: h) of a broad, approximately 3–10 kDa interval of immunoreactive fractions. PGH peptides were revealed as different MW forms, especially in the hypothalamus (Fig. 6, right). The largest MW forms eluting in the void volume were low in tissues and abundant in plasma (ibidem: i). Interestingly, an intermediate MW peak (ibidem: m, roughly 30–50 kDa) might correspond to PGH peptide/s encompassing the whole portion between the PGH sequence and either of the known cleaved rat peptides: VGF_180–209_, or VGF_24–36_ [31]; [32], see (Fig. 1). The low MW peak shown (ibidem: n) fits well with the reported PGH8 peptide [33], running between the PGH sequence and the immediately preceding cleavage site (rat VGF: Lys_421_-Arg_422_).

### *In vitro* experiments

**Figure 7:**
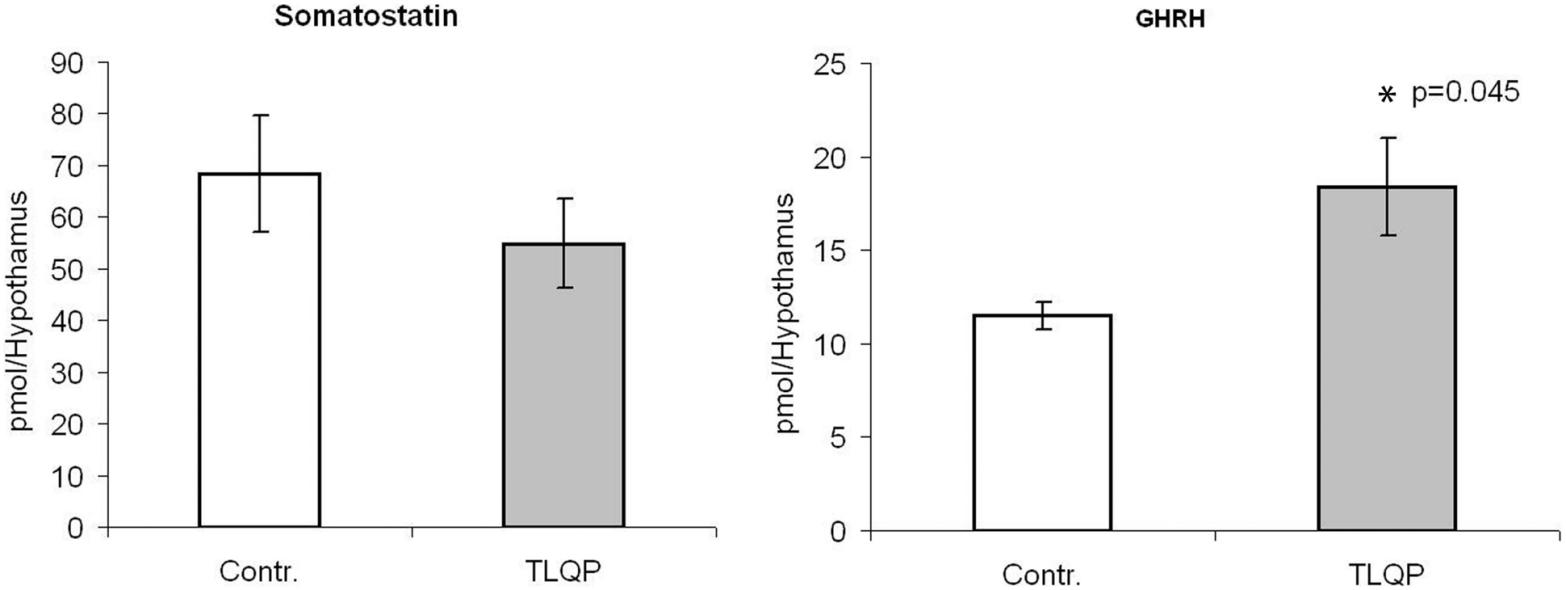
TLQP-21 in *in vitro* experiments. TLQP-21 included in the medium of hypothalamic samples up-regulates the secretion of growth-hormone-releasing hormone (GHRH). Contr.: control medium analysed by ELISA without the addition of TLQP-21 *versus* the medium including the peptide (TLQP column).

A single dose of TLQP-21 was tested, comparable to the one shown to induce release of GnRH from male rat hypothalami [24]. Upon addition of TLQP-21 to the culture medium, somatostatin release into the medium showed a non-significant tendency to decrease (Fig. 7, left panel). Conversely, GHRH release was significantly enhanced (approximately 50%, Fig. 7, right panel).

## Discussion

Our study demonstrated the presence of several peptides derived from the VGF precursor, with selective differential profiles at all levels of the hypothalamic-pituitary-ovarian axis. A striking modulation in peptide tissue levels including plasma was shown across the estrous cycle, implicating VGF peptides in events and mechanisms relevant to reproduction.

In view of the proposed role of VGF as multifunctional precursor of bioactive peptides our study was focused on end products of the VGF precursor, rather than on the primary VGF gene product itself. The primary sequence of VGF shows at least ten putative cleavage sites, in the form of two or more basic amino acid residues, highly conserved across species. Recently, further fragments including bioactive VGF peptides have been shown to derive from cleavage at single basic amino acids [16], [34], [35]. In view of the lower proteomic complexity of the cerebro-spinal fluid (CSF), the precise chemical nature of VGF derived end products has so far been mostly studied in such body fluid [36] with the addition of neuro-endocrine cells lines [33]. An array of VGF peptides and fragments have been revealed, with striking changes in neurodegenerative and other disease conditions [37]–[40]. Certain VGF peptides proved to be released *in vivo* in the brain upon neuronal depolarization [31] while an interactomic investigation revealed selective domains of VGF, including the TLQP-21 region, as likely binding partners for the amyloid precursor protein [41]. Similarly, two peptides derived from non-overlapping regions of the VGF precursor were reported in the protein-bound and un-bound fractions, respectively, from CSF [42]. In the present study, TLQP-antibodies showed several chromatography peaks fitting well with known VGF peptides, such as TLQP-21, -30 and -62 [30]. Products derived from the C-terminal domain of VGF have been studied in some detail [11], [12] and the major molecular form/s we found in hypothalamus and pituitary may represent so-called VGF18 and 20 (such denomination refers to migration in western blot, not to MW). In connection with the TLQP-62 peak shown here with the relevant N-terminal antibody (“TLQP rat 556–565” antibody), a lower amount of reactivity was revealed in chromatography with the corresponding C-terminus antibody. The very high selectivity of such antibody that we found for the unmodified C-terminal sequence suggests that TLQP-62 peptide/s may partly undergo limited processing at their C-terminal end. Other peptides derived from the C-terminal domain of the VGF precursor may have gone undetected in our study, due to a degree of processing or change at their C-terminus. At the other extreme of VGF, a fully N-terminal 40 amino acid peptide has been proved to be released upon neuronal depolarization [31]. Such peptide may take part in a broad region of immunoreactivity we found in the hypothalamus and probably elsewhere. A small PGH peptide (“PGH8”), corresponding to a sequence running from the C-terminal peptide sequence we used to raise antibodies (“PGH rat_422–430_” antibody), to the immediately preceding putative cleavage site (Lys-Arg, rat VGF_421–422_, “R” preceding the “PGH rat 422–430”) was demonstrated in mouse neuroblastoma cells [33]. While several intermediate MW PGH peptides are difficult to interpret, one may speculate that the broad immunoreactive peak close to the void volume that we found in gel chromatography could include putative, large VGF fragments running from the PGH sequence to a region close to the N-terminus of VGF. In plasma, we revealed certain VGF peptides possibly including TLQP-21, TLQP-30 and TLQP-62, however, in general, a large MW peak was prominent with all VGF assays. This may indicate that one or more VGF peptides travel in the circulation bound to plasma proteins. In fact, many polypeptides are known to bind to major plasma proteins, and/or to their selective transporter/s. Concerning to our study, one ought to notice that tissue extracts underwent at least some treatment steps (e.g. heat tratment) which would reduce molecular interactions of VGF peptides with other molecules, while plasma samples was neither extracted, nor treated before assays. While examples of the binding of VGF peptides to other components are provided above, the possibility that at least some of the VGF-immunoreactive large MW forms found in plasma may represent actual VGF precursor cannot be excluded.

On this context, the selective differential MW profiles of the VGF peptides were found in parallel with a striking modulation in their tissue levels across the estrous cycle.

With respect to the TLQP peptides, as mentioned, they were found within median eminence somatostatin neurones, gonadotropic and ovary cells with high tissue levels especially in ovary and plasma. In the blood, their low levels in ovariectomised rats showed the ovary as a major source of circulating TLQP peptides. During the cycle, we showed an increase in pituitary TLQP levels on metestrus–diestrus. This is according to the increase in LH secretion obtained after TLQP-21 *icv* administration in adult female rats on the same diestrous phase [25]. Moreover, we reported here that, on proestrus, the TLQP ovary levels were augmented, but during the following phases (including the diestrus), the same ovary reduced its TLQP peptide content that concurrently augmented in blood. The possible involvement of TLQP peptides in endocrine mechanisms related to reproduction is suggested also by the reported increase in LH secretion after system *ip* administration of TLQP-21 on prepubertal rats [25]. Hence, from our results, one may hypothesize that during the diestrus-metestrus, circulating TLQP peptides secreted from the ovary, may stimulate the pituitary secretion of LH, perhaps through an augment of the same TLQP content in the pituitary. The involvement of TLQP peptides on female reproductive mechanisms has so far been reported to be related to the LH production at the pituitary [25] but not at the gonad level. As to the potential TLQP receptors, the same C3a/C3AR1 molecules that have been found to modulate the pituitary hormonal activity [43] have been reported to be the targets of the TLQP-21 in the hamster ovary cell lines [44]. Hence, the same ovary that secretes TLQP peptides into the blood could be regulated by its own derived peptides, or alternatively the circulating TLQP peptides reacting to the ovary receptors could originate, at least in part, from the hypophysis. This is suggested by our finding in estrous phase, where a marked reduction in TLQP pituitary levels (according to a minor number of immnolabeled cells) was found in parallel with a TLQP plasmatic increase.

Moreover, we cannot exclude the possibility that TLQP peptides could also act through a paracrine and/or autocrine way, in view of their presence in the same organs (pituitary and ovary) that contain their potential receptors. This autocrine/paracrine role could involve the regulation of LH secretion, according to the mentioned augment in TLQP pituitary levels on the same phase in which the LH secretion was found to increase [25]. Indeed, an autocrine role have been already demonstrated for other VGF peptides as NERPs that being present within the vasopressin cells, regulated the same vasopressin production [16], [34]. On the other end, our results showed also that TLQP peptides in the pituitary and hypothalamus could be up-regulated by the oestradiol/progesterone.

These hormones regulate also somatostatin [45], and being TLQP peptides present in such neurones, it is possible that steroid hormones could modulate both TLQP and somatostatin by the same mechanisms. Regarding the role of TLQP peptides in the median eminence, in view of their close relation to somatostatin seen also in pancreas [46] and stomach [30], we tried to elucidate a possible bioactivity of TLQP-21 on somatostatin and on its GHRH neurone targets. We found that TLQP-21 up-regulated the GHRH production suggesting that such peptide could act promoting the growth at the hypothalamic level. When adult male mice were chronically treated (by *icv* injection) with TLQP-21, physiological, molecular and behavioral parameters related to the GH axis were investigated, however, TLQP-21 did not modulate the GH axis [47]. One could hypothesize that TLQP-21 may regulate GHRH through somatostatin neurones selectively at the median eminence level, instead when the same peptide is injected, only a little part could reach the median eminence. Further experiments are needed to ascertain the precise role of TLQP-21 on the growth mechanisms and the possible connections with the cycle. Regarding the other VGF peptides, also C-and N-terminus peptides changed in the hypothalamus, pituitary and plasma. However, for these peptides is more difficult to hypothesize mechanisms of action in view of the rare information regarding their involvement on reproduction. It could be only suggested that these peptides could have a neuroendocrine and/or endocrine activity on the estrous cycle, and their presence into kisspeptin neurones is intriguing because of the importance of this hormone in reproductive mechanisms [48], [49]. Furthermore, also the presence of PGH peptides in gonadotrophs, GnRH neurones, plasma and especially within the oocyte, needs more investigation. In conclusion, various VGF peptides may regulate the hypothalamus-pituitary complex *via* specific neuroendocrine mechanisms. In particular, for TLQP peptides we may hypothesize different possible interacting ways of action on the reproduction including endocrine pathways largely involving the ovary as well as paracrine/autocrine mechanisms at both the pituitary and ovary levels.

In addition, we also found evidence for an involvement of TLQP-21 on neuroendocrine mechanisms acting on promoting growth at the median eminence level.

## Supporting Information

Data S1 **TLQP and C-terminus assay results.** For each assay, data referring to picomoles per gram or milliliter revealed by ELISA for each single animal case in each tissue tested through the 4 cycle phases.
(TIFF)

Data S2 **N-terminus and PGH assay results.** For each assay, data referring to picomoles per gram or milliliter revealed by ELISA for each single animal case in each tissue tested through the 4 cycle phases.
(TIFF)

Data S3 **TLQP, C-terminus, N-terminus and PGH assay results.** For each assay, data referring to picomoles per gram and milliliter revealed by ELISA for each single animal case in each tissue tested using ovariectomised rats without any hormonal tratment (OVX) and treated with estrogen-progesterone (OVX + EP).
(TIFF)

Data S4 **Somatostatin and GHRH assay results.** For each assay, data referring to picomoles per well, grams per well, total grams of hypothalamus, as well as picomoles per hypothalamus and milliliter obtained using each single hypothalamic sample with (TLQP: B1 to B8) and without (contr: controls, A1 to A6) the addition of the TLQP-21 peptide to the culture medium.
(TIFF)

## References

[1] Hahm S, Mizuno TM, Wu TJ, Wisor JP, Priest CA, et al (1999) Targeted deletion of the vgf gene indicates that the encoded secretory peptide precursor plays a novel role in the regulation of energy balance. Neuron 23: 537–548. 10433265 10.1016/s0896-6273(00)80806-5

[2] Levi A, Eldridge JD, Paterson BM (1985) Molecular cloning of a gene sequence regulated by nerve growth factor. Science 229: 393–5. 3839317 10.1126/science.3839317

[3] Salton SR, Fischberg DJ, Dong KW (1991) Structure of the gene encoding VGF, a nervous system-specific mRNA that is rapidly and selectively induced by nerve growth factor in PC12 cells. Mol Cell Biol 11(5): 2335–2349. 2017159 10.1128/mcb.11.5.2335-2349.1991 PMC359984

[4] Snyder SE, Pintar JE, Salton SR (1998) Developmental expression of VGF mRNA in the prenatal and postnatal rat. J Comp Neurol 394: 64–90. 9550143

[5] Snyder SE, Salton SR (1998) Expression of VGF mRNA in the adult rat central nervous system. J Comp Neurol 394(1): 91–105. 9550144

[6] Van den Pol AN, Decavel C, Levi A, Paterson B (1989) Hypothalamic expression of a novel gene product, VGF: immunocytochemical analysis. J Neurosci 9: 4122–4137. 2556505 10.1523/JNEUROSCI.09-12-04122.1989 PMC6569627

[7] Van den Pol AN, Bina K, Decavel C, Ghosh P (1994) VGF expression in the brain. J Comp Neurol 347: 455–469. 7822494 10.1002/cne.903470311

[8] Levi A, Ferri GL, Watson E, Possenti R, Salton SR (2004) Processing, distribution, and function of VGF, a neuronal and endocrine peptide precursor. Cell Mol Neurobiol: 24 517−33. 10.1023/B:CEMN.0000023627.79947.22 PMC11529936 15233376

[9] Salton SR, Ferri GL, Hahm S, Snyder SE, Wilson AJ, et al (2000) VGF: a novel role for this neuronal and neuroendocrine polypeptide in the regulation of energy balance. Front Neuroendocrinol 21: 199–219. 10882540 10.1006/frne.2000.0199

[10] Possenti R, Rinaldi AM, Ferri GL, Borboni P, Trani E, et al (1999) Expression, processing, and secretion of the neuroendocrine VGF peptides by INS-1 cells. Endocrinology 140: 3727–3735. 10433233 10.1210/endo.140.8.6920

[11] Trani E, Ciotti T, Rinaldi AM, Canu N, Ferri GL, et al (1995) Tissue-specific processing of the neuroendocrine protein VGF. J Neurochem 65: 2441–2449. 7595538 10.1046/j.1471-4159.1995.65062441.x

[12] Trani E, Giorgi A, Canu N, Amadoro G, Rinaldi AM, et al (2002) Isolation and characterization of VGF peptides in rat brain. Role of PC1/3 and PC2 in the maturation of VGF precursor. J Neurochem 81: 565–574. 12065665 10.1046/j.1471-4159.2002.00842.x

[13] Canu N, Possenti R, Rinaldi AM, Trani E, Levi A (1997) Molecular cloning and characterization of the human VGF promoter region. J Neurochem 68: 1390–1399. 9084409 10.1046/j.1471-4159.1997.68041390.x

[14] Bartolomucci A, La Corte G, Possenti R, Locatelli V, Rigamonti AE, et al (2006) TLQP-21, a VGF-derived peptide, increases energy expenditure and prevents the early phase of diet-induced obesity. Proc Natl Acad Sci U S A 103(39): 14584–14589. 16983076 10.1073/pnas.0606102103 PMC1600003

[15] Bozdagi O, Rich E, Tronel S, Sadahiro M, Patterson K, et al (2008) The neurotrophin-inducible gene vgf regulates hippocampal function and behavior through a brain-derived neurotrophic factor-dependent mechanism. J Neurosci 28: 9857–69. 18815270 10.1523/JNEUROSCI.3145-08.2008 PMC2820295

[16] Yamaguchi H, Sasaki K, Satomi Y, Shimbara T, Kageyama H, et al (2007) Peptidomic identification and biological validation of neuroendocrine regulatory peptide-1 and -2. J Biol Chem 282: 26354–26360. 17609209 10.1074/jbc.M701665200

[17] Rizzi R, Bartolomucci A, Moles A, D’Amato F, Sacerdote P, et al (2008) The VGF-derived peptide TLQP-21: a new modulatory peptide for inflammatory pain. Neurosci Lett 441: 129–133. 18586396 10.1016/j.neulet.2008.06.018

[18] Chen YC, Pristerá A, Ayub M, Swanwick RS, Karu K, et al.. (2013) Identification of a receptor for neuropeptide VGF and its role in neuropathic pain. J Biol Chem: 288 34638−34646. 10.1074/jbc.M113.510917 PMC3843076 24106277

[19] Razzoli M, Bo E, Pascucci T, Pavone F, D’Amato FR, et al (2012) Implication of the VGF-derived peptide TLQP-21 in mouse acute and chronic stress responses. Behav Brain Res 229(2): 333–339. 22289198 10.1016/j.bbr.2012.01.038

[20] Severini C, La Corte G, Improta G, Broccardo M, Agostini S, et al (2009) In vitro and in vivo pharmacological role of TLQP-21, a VGF-derived peptide, in the regulation of rat gastric motor functions. Br J Pharmacol. 157(6): 984–93. 10.1111/j.1476-5381.2009.00192.x PMC2737657 19466987

[21] Ferri GL, Gaudio RM, Cossu M, Rinaldi AM, Polak JM, et al (1995) The VGF protein in rat adenohypophysis: sex differences and changes during the estrous cycle and after gonadectomy. Endocrinology 136: 2244–2251. 7720674 10.1210/endo.136.5.7720674

[22] Brancia C, Nicolussi P, Cappai P, La Corte G, Possenti R, et al (2005) Differential expression and seasonal modulation of VGF peptides in sheep pituitary. J Endocrinol 186: 97–107. 16002540 10.1677/joe.1.05992

[23] Babbitt CC, Tung J, Wray GA, Alberts SC (2012) Changes in gene associated with reproductive maturation in wild female baboons. Genome Biol Evol 4(2): 102–109. 22155733 10.1093/gbe/evr134 PMC3273164

[24] Pinilla L, Pineda R, Gaytán F, Romero M, García-Galiano D, et al (2011) Characterization of the reproductive effects of the anorexigenic VGF-derived peptide TLQP-21: in vivo and in vitro studies in male rats. Am J Physiol Endocrinol Metab 300(5): E837–847. 21304062 10.1152/ajpendo.00598.2010

[25] Aguilar E, Pineda R, Gaytán F, Sánchez-Garrido MA, Romero M, et al (2013) Characterization of the reproductive effects of the VGF-derived peptide, TLQP-21, in female rats: in vivo and in vitro studies. Neuroendocrinology 98(1): 38–50. 23485923 10.1159/000350323

[26] Cocco C, Melis GV, Ferri GL (2003) Embedding media for cryomicrotomy: an applicative reappraisal. Appl Immunohistochem Mol Morphol 11(3): 274–80. 12966356 10.1097/00129039-200309000-00012

[27] Ferri GL, Cocco C, Melis GV, Aste L (2002) Equipment testing and tuning: the cold-knife cryomicrotome microm HM-560. Appl Immunohistochem Mol Morphol 10(4): 381–386. 12607609 10.1097/00129039-200212000-00016

[28] D’Amato F, Noli B, Brancia C, Cocco C, Flore G, et al (2008) Differential distribution of VGF-derived peptides in the adrenal medulla and evidence for their selective modulation. J Endocrinol 197: 359–369. 18434366 10.1677/JOE-07-0346

[29] Cocco C, D’Amato F, Noli B, Ledda A, Brancia C, et al (2010) Distribution of VGF peptides in the human cortex and their selective changes in Parkinson’s and Alzheimer’s diseases. J Anat 217: 683–93. 21039478 10.1111/j.1469-7580.2010.01309.x PMC3039181

[30] Brancia C, Cocco C, D’Amato F, Noli B, Sanna F, et al (2010) Selective expression of TLQP-21 and other VGF peptides in gastric neuroendocrine cells and modulation by feeding. J Endocrinol 207: 329–341. 20876237 10.1677/JOE-10-0189

[31] Bernay B, Gaillard M-C, Guryca V, Emadali A, Kuhn L, et al (2009) Discovering New Bioactive Neuropeptides in the Striatum Secretome Using in Vivo Microdialysis and Versatile Proteomics. Mol Cell Proteomics 8(5): 946–58. 19164277 10.1074/mcp.M800501-MCP200 PMC2689773

[32] Sasaki K, Takahashi N, Satoh M, Yamasaki M, Minamino N (2010) A Peptidomics Strategy for Discovering Endogenous Bioactive Peptides. Journal of Proteome Research 9: 5047–5052. 20681733 10.1021/pr1003455

[33] Rozek W, Kwasnik M, Debski J, Zmudzinski JF (2013) Mass spectrometry identification of granins and other proteins secreted by neuroblastoma cells. Tumor Biol 34: 1773–1781. 10.1007/s13277-013-0716-0 PMC3661923 23519838

[34] Fujihara H, Sasaki K, Mishiro-Sato E, Ohbuchi T, Dayanithi G, et al (2012) Molecular Characterization and Biological Function of Neuroendocrine Regulatory Peptide-3 in the Rat. Endocrinology 153(3): 1377–86. 22253422 10.1210/en.2011-1539

[35] Sasaki K, Osaki T, Minamino N (2012) Large-scale identification of endogenous secretory peptides using electron transfer dissociation mass spectrometry. Mol Cell Proteomics 12(3): 700–9. 23250050 10.1074/mcp.M112.017400 PMC3591662

[36] Yuan X, Desiderio DM (2005) Human cerebrospinal fluid peptidomics. J Mass Spectr 40: 176–181. 10.1002/jms.737 15706611

[37] Carrette O, Demalte I, Scherl A, Yalkinoglu O, Corthals G, et al (2003) A panel of cerebrospinal fluid potential biomarkers for the diagnosis of Alzheimer’s disease. Proteomics 3: 1486–1494. 12923774 10.1002/pmic.200300470

[38] Ruetschi U, Zetterberg H, Podust VN, Gottfries J, Li S, et al (2005) Identification of CSF biomarkers for frontotemporal dementia using SELDI-TOF. Experimental Neurology 196: 273–281. 16154129 10.1016/j.expneurol.2005.08.002

[39] Busse S, Bernstein HG, Busse M, Bielau H, Brisch R, et al (2012) Reduced density of hypothalamic VGF-immunoreactive neurons in schizophrenia: a potential link to impaired growth factor signaling and energy homeostasis. Eur Arch Psychiatry Clin Neurosc 262(5): 365–74. 10.1007/s00406-011-0282-7 22167530

[40] Asano T, Koizumi S, Takagi A, Hatori T, Kuwabara K, et al (2011) Identification of a novel biomarker candidate, a 4.8-kDa peptide fragment from a neurosecretory protein VGF precursor, by proteomic analysis of cerebrospinal fluid from children with acute encephalopathy using SELDI-TOF-MS. Neurology 11: 101. 21838886 10.1186/1471-2377-11-101 PMC3174120

[41] Bai Y, Markham K, Chen F, Weerasekera R, Watts J, et al (2005) The in vivo brain interactome of the amyloid precursor protein. Mol Cell Proteomics (1): 15–34. 10.1074/mcp.M700077-MCP200 17934213

[42] Wijte D (2012) McDonnell LA, Balog CI, Bossers K, Deelder AM, et al (2012) A novel peptidomics approach to detect markers of Alzheimer’s disease in cerebrospinal fluid. Methods 56(4): 500–7. 22465281 10.1016/j.ymeth.2012.03.018

[43] Francis K, Lewis BM, Akatsu H, Monk PN, Cain SA (2003) Complement C3a receptors in the pituitary gland: a novel pathway by which an innate immune molecule releases hormones involved in the control of inflammation. FASEB J 17(15): 2266–8. 14563692 10.1096/fj.02-1103fje

[44] Hannedouche S, Beck V, Leighton-Davies J, Beibel M, Roma G, et al (2013) Identification of the C3a receptor (C3AR1) as the target of the VGF-derived peptide TLQP-21 in rodent cells. J Biol Chem 288(38): 27434–27443. 23940034 10.1074/jbc.M113.497214 PMC3779738

[45] Van Vugt HH, Van de Heijning BJ, Van der Beek EM (2008) Somatostatin in the rat periventricular nucleus: sex differences and effect of gonadal steroids. Exp Brain Res 188(4): 483–491. 18421448 10.1007/s00221-008-1381-1 PMC2441535

[46] Cocco C, Brancia C, Pirisi I, D’Amato F, Noli B, et al (2007) VGF metabolic-related gene: distribution of its derived peptides in mammalian pancreatic islets. J Histochem Cytochem 55: 619–628. 17312015 10.1369/jhc.6A7040.2007

[47] Bartolomucci A, Rigamonti AE, Bulgarelli I, Torsello A, Locatelli V, et al (2007) Chronic intracerebroventricular TLQP-21 delivery does not modulate the GH/IGF-1-axis and muscle strength in mice. Growth Horm IGF Res 17(4): 342–5. 17400498 10.1016/j.ghir.2007.02.002

[48] Navarro VM, Castellano JM, Fernández-Fernández R, Tovar S, Roa J, et al (2005) Effects of KiSS-1 peptide, the natural ligand of GPR54, on follicle-stimulating hormone secretion in the rat. Endocrinology 146(4): 1689–1697. 15637288 10.1210/en.2004-1353

[49] Adachi S, Yamada S, Takatsu Y, Matsui H, Kinoshita M, et al (2007) Involvement of anteroventral periventricular metastin/kisspeptin neurons in estrogen positive feedback action on luteinizing hormone release in female rats. J Reprod Dev 53(2): 367–78. 17213691 10.1262/jrd.18146

