# Supplementary figures and images for: VGF Changes during the Estrous Cycle: A Novel Endocrine Role for TLQP Peptides?
Source: PLoS One. 2014 Oct 3;9(10):e108456. doi: 10.1371/journal.pone.0108456 (PMC4184793; doi:10.1371/journal.pone.0108456)

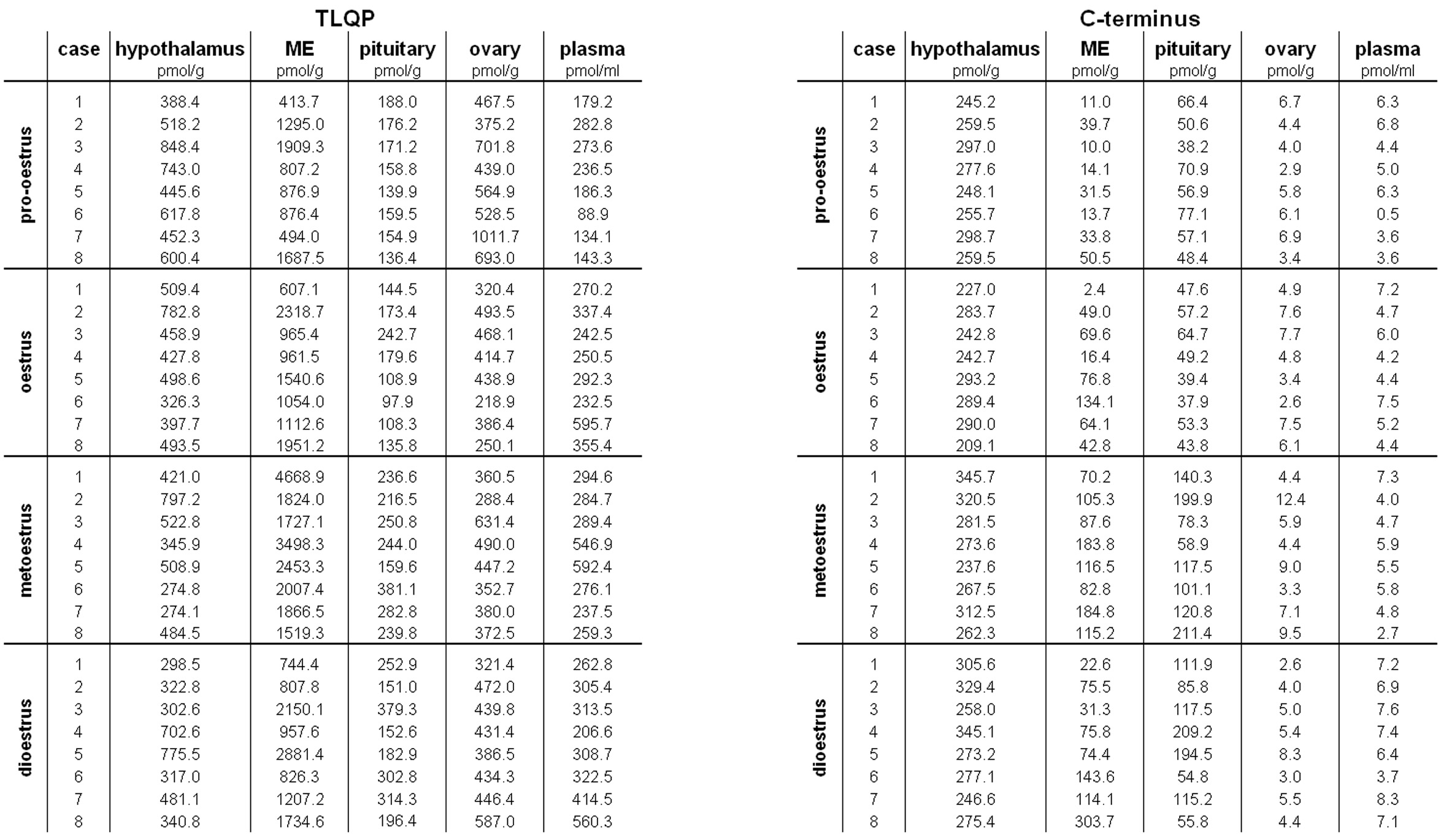

Supplement: Data S1 — TLQP and C-terminus assay results. For each assay, data referring to picomoles per gram or milliliter revealed by ELISA for each single animal case in each tissue tested through the 4 cycle phases. (TIFF) [file pone.0108456.s001.tiff]

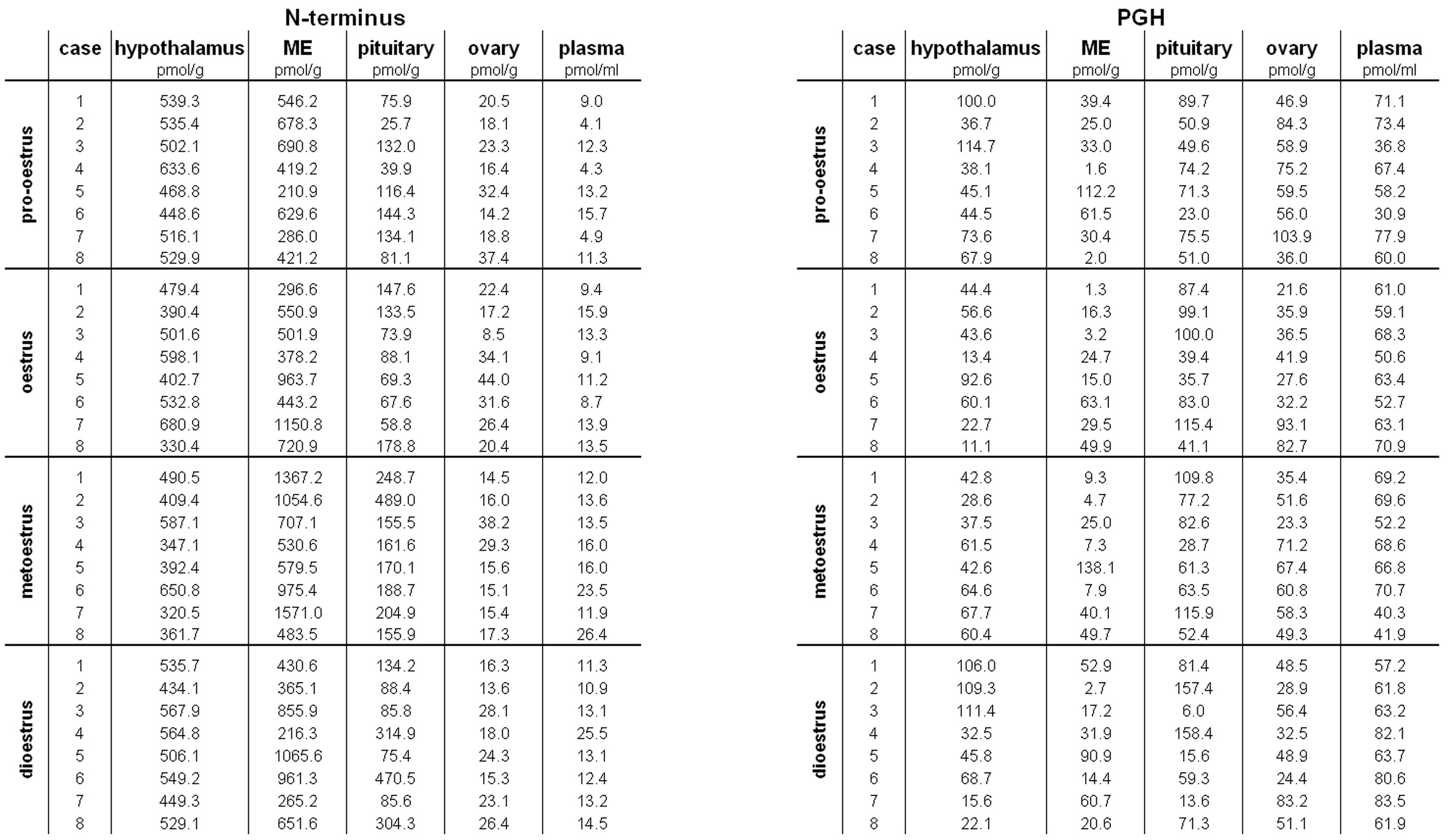

Supplement: Data S2 — N-terminus and PGH assay results. For each assay, data referring to picomoles per gram or milliliter revealed by ELISA for each single animal case in each tissue tested through the 4 cycle phases. (TIFF) [file pone.0108456.s002.tiff]

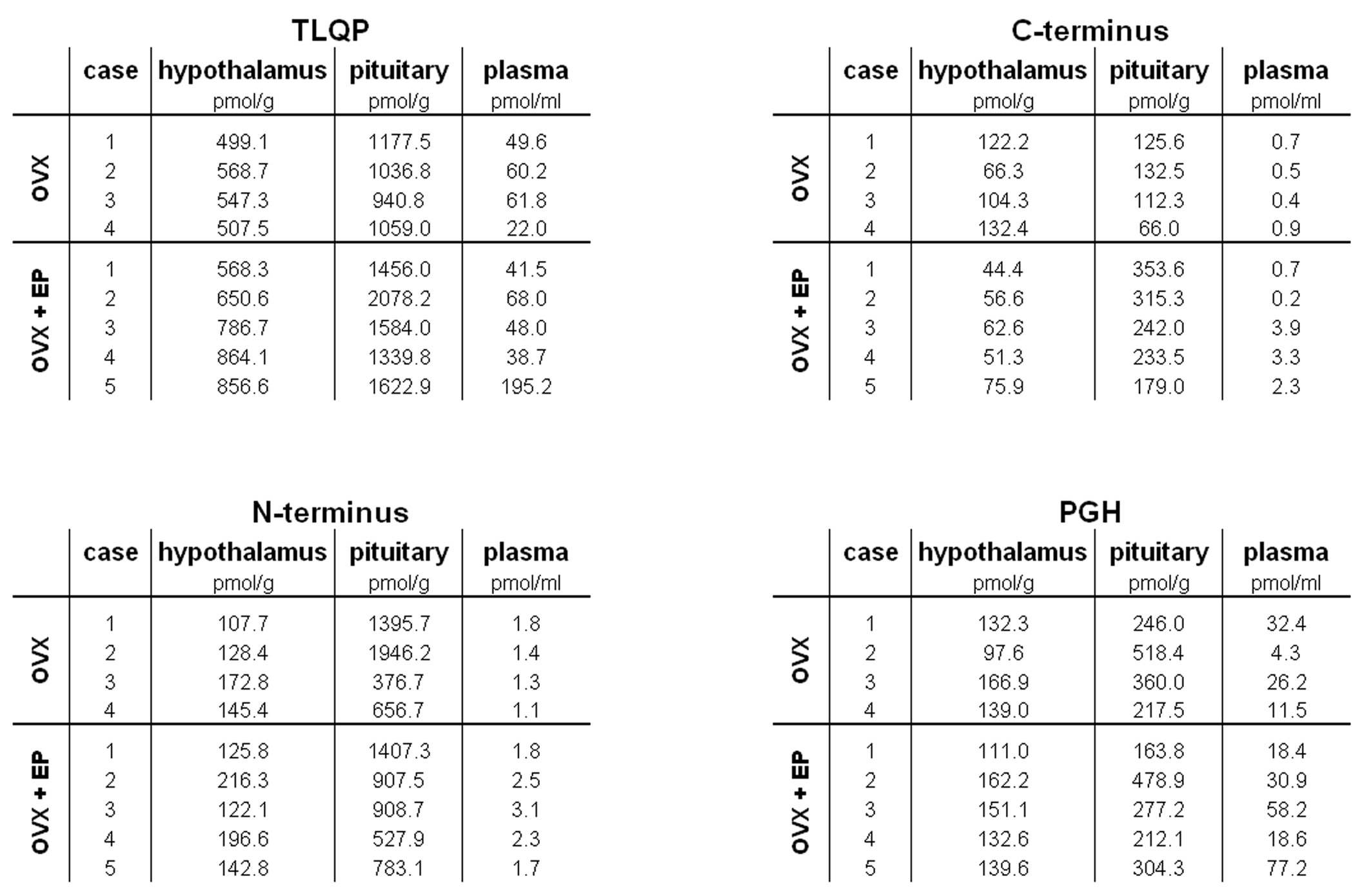

Supplement: Data S3 — TLQP, C-terminus, N-terminus and PGH assay results. For each assay, data referring to picomoles per gram and milliliter revealed by ELISA for each single animal case in each tissue tested using ovariectomised rats without any hormonal tratment (OVX) and treated with estrogen-progesterone (OVX + EP). (TIFF) [file pone.0108456.s003.tiff]

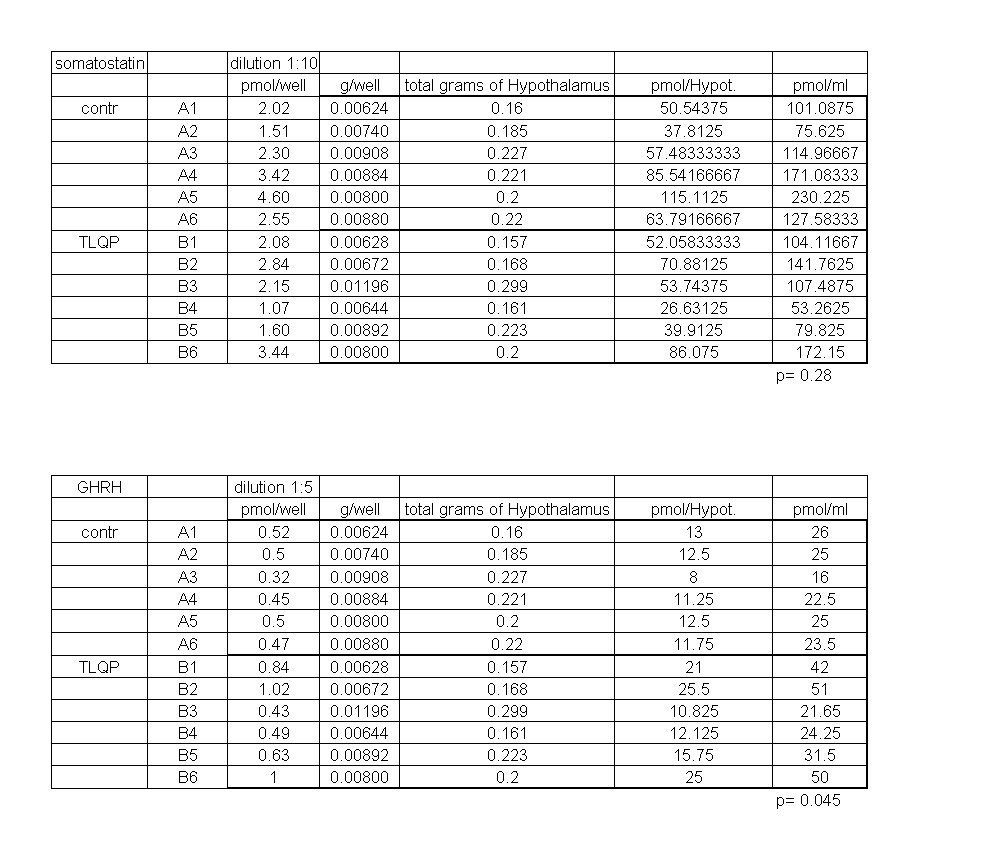

Supplement: Data S4 — Somatostatin and GHRH assay results. For each assay, data referring to picomoles per well, grams per well, total grams of hypothalamus, as well as picomoles per hypothalamus and milliliter obtained using each single hypothalamic sample with (TLQP: B1 to B8) and without (contr: controls, A1 to A6) the addition of the TLQP-21 peptide to the culture medium. (TIFF) [file pone.0108456.s004.tiff]
